# Inequalities in mammography and Papanicolaou test coverage: a time-series study

**DOI:** 10.1590/1516-3180.2020.0166.R1.02092020

**Published:** 2020-10-26

**Authors:** Deborah Carvalho Malta, Elton Junio Sady Prates, Alanna Gomes da Silva, Filipe Malta dos Santos, Greice de Campos Oliveira, Nádia Machado de Vasconcelos, Elier Broche Cristo

**Affiliations:** I MD, PhD. Associate Professor, Postgraduate Program of the School of Nursing, Universidade Federal de Minas Gerais (UFMG), Belo Horizonte (MG), Brazil.; II Undergraduate Nursing Student, School of Nursing, Universidade Federal de Minas Gerais (UFMG), Belo Horizonte (MG), Brazil.; III MSc. Nurse and Doctoral Student, Postgraduate Program of the School of Nursing, Universidade Federal de Minas Gerais (UFMG), Belo Horizonte (MG), Brazil.; IV MD. Master's Student, Public Health Postgraduate Program of the School of Medicine, Universidade Federal de Minas Gerais (UFMG), Belo Horizonte (MG), Brazil.; V MD. Medical Resident, Contagem Mother and Child Center, Contagem (MG), Brazil.; VI MD. Master's Student, Public Health Postgraduate Program of the School of Medicine, Universidade Federal de Minas Gerais (UFMG), Belo Horizonte (MG), Brazil.; VII MSc. Systems Engineer, Health Surveillance Department, Ministry of Health, Brasília (DF), Brazil.

**Keywords:** Mammography, Papanicolaou test, Risk factors, Health status disparities, Noncommunicable diseases, Breast cancer, Cervical cancer, Surveillance, Screening test, Women's health, Secondary prevention

## Abstract

**BACKGROUND:**

Cancer is a serious public issue problem worldwide. In Brazil, breast cancer is the most common type and cervical cancer is the third most frequent among women.

**OBJECTIVE:**

To analyze the temporal trend of coverage of mammography and cervical oncotic cytological testing, between 2007 and 2018.

**DESIGN AND SETTING:**

Time-series study conducted in the 26 Brazilian state capitals and in the Federal District.

**METHODS:**

A linear regression model was used to estimate trends in coverage of mammography and cervical oncotic cytological testing over the period. The data collection system for Surveillance of Risk and Protection Factors for Chronic Diseases by Telephone Survey (Vigitel) was used.

**RESULTS:**

A significant increase in mammography coverage was observed, from 71.1% in 2007 to 78.0% in 2018. There was a trend towards an increase among women with 0 to 8 years of schooling, in all regions of Brazil. Regarding cervical oncotic cytological testing coverage, there was a trend towards stability during the period analyzed, reaching 81.7% in 2018. On the other hand, there was a significant increase in the northern region.

**CONCLUSIONS:**

There was an improvement in the coverage of these screening examinations, especially regarding mammography. However, it is still necessary to expand their provision, quality and surveillance, aimed towards women's health.

## INTRODUCTION

Cancer is a serious public health issue worldwide. In Brazil, among women, the most common type is breast cancer (29.7%) and cervical cancer is the third most frequent (7.4%).^1^ In 2017, there were 16,724 deaths from breast cancer and 6,385 from cervical cancer.^1^ They were responsible, respectively, for the losses of 551,306.08 and 59,498.97 million disability-adjusted life years (DALYs).^2^ There are also great variations in the magnitude and types of cancer across the different regions of Brazil.^1^

Brazil is expected to have 66,280 cases of breast cancer diagnosed per year between 2020 and 2022, corresponding to a rate of 61.6 diagnoses per 100,000 women. The number of new cervical cancer cases expected for the same period would be 16,590, corresponding to a rate of 15.43 per 100,000 women.^1^

Cervical and breast cancer incidence, mortality and morbidity may be reduced through effective control strategies. These should include screening programs, health promotion actions, prevention, early diagnosis, treatment, rehabilitation and palliative care, when necessary.^3^

The Brazilian National Health System (Sistema Único de Saúde, SUS) guarantees universal free access to mammography examinations and cervical cytological testing, also known as the Papanicolaou test. The Brazilian Ministry of Health recommends screening mammography for women aged 50 to 69, to be done every two years.^4^

The screening method for cervical cancer and its precursor lesions is oncotic cytological testing. Screening should start at the age of 25 for women who have already had sexual activity and periodic examinations must continue until they are 64 years old. The first two examinations should be performed at an annual interval and, if both results presented satisfactory samples and were negative for malignancy, subsequent examinations should be performed every three years.^5^

To promote development and implementation of effective, integrated, sustainable and evidence-based public policies, the federal government launched the Strategic Action Plan for Confronting Chronic Noncommunicable Diseases in Brazil, 2011-2022. Among the proposed national targets were increases in mammography coverage among women between 50 and 69 years old to 70% and in Papanicolaou test coverage among women from 25 to 64 years old to 85%; promotion of improved quality of screening tests; and treatment of 100% of women diagnosed with precursor cancer lesions. Among the actions to speed up the diagnosis, there were investments in diagnostic capacity and infrastructure, especially in the northern and northeastern regions of Brazil.^6^^,^^7^

## OBJECTIVE

Thus, the objective of the present study was to analyze the temporal trends of mammography and cervical oncotic cytological test coverage, between the years 2007 and 2018.

## METHODS

### Study design and data collection

This study analyzed the trends in coverage of mammography and cervical oncotic cytological tests using data covering the years between 2007 and 2018 that were collected from the Surveillance of Risk and Protection Factors for Chronic Diseases by Telephone Survey (Vigitel).

Vigitel is a survey conducted through telephone calls in the Brazilian population, which annually monitors the main chronic noncommunicable diseases (NCDs) and their risk and protection factors. This survey is conducted on a representative sample of the adult population in Brazil (≥ 18 years old) living in households with at least one fixed telephone line, in each of the 26 Brazilian state capitals and in the Federal District. Every year, approximately 2,000 people answer the survey questions and, over the years in which Vigitel has been conducted, 382,255 adult women have been interviewed. Survey professionals have applied some adjustment procedures that have taken sex, age and education levels into account, with the aim of reducing the non-representation bias inherent to telephone interviews and seeking to make the sample distribution similar to the sociodemographic characteristics of the adult population of each state capital.^8^ Details on the sampling and data collection process can be found in the published Vigitel results.^8^^,^^9^

### Indicator definition

The mammography and cervical oncotic cytological testing coverage indicators used in the study were obtained through the following Vigitel questions:^8^

- **Percentage of women (50 to 69 years old) who underwent mammography examinations over the last two years:** a measurement of the number of women between 50 and 69 years old who underwent mammography over the last two years, derived from the number of women between these ages who were interviewed. This was in answer to the questions: “Did you ever have a mammogram breast x-ray?” and “How long ago did you have a mammogram?”. These questions were only applied to women between 50 and 69 years of age because this is the age range within which breast cancer screening through mammography is recommended.
- **Percentage of women (25 to 64 years old) who underwent a Papanicolaou test for cervical cancer over the last three years:** a measurement of the number of women between 25 and 64 years old who underwent an oncotic cytological examination over the last three years, derived from the number of women between these ages who were interviewed. This was in answer to the questions: “Did you ever have a Papanicolaou test/cervical cancer screening?” and “How long has it been since you took a Papanicolaou test?”

### Statistical analysis

The indicators were stratified according to schooling level (0 to 8; 9 to 11; and ≥ 12 years), Brazilian state capitals and regions (North, Northeast, Southeast, South and Center-West) and age groups for the Papanicolaou test (25 to 34; 35 to 44; 45 to 54; and 55 to 64 years) and mammography (50 to 59; and 60 to 69 years).

The dependent variables were the prevalences of mammography and cervical cytological test coverage and the independent variable was the year of the survey.

A linear regression model was used to estimate trends over the period. Significant linear trends were considered to exist when the slope of the model was different from zero for a P-value ≤ 0.05. The adjusted R^2^ value was used as a measurement of model fit.

The analyses were performed using the Stata software (Stata Corp LP, College Station, United States), version 13.0. Quantum GIS (QGIS) version 3.12.0 (QGIS.org (2020); QGIS Geographic Information System; Open Source Geospatial Foundation Project) was used to build the maps.

The Vigitel data is available for public access and use. The National Commission for Research Ethics of the Ministry of Health approved collection of these survey data on human beings (number: 355.590; date: June 26, 2013). Informed consent was obtained orally, at the time of telephone contact with the interviewees.

## RESULTS

Over the entire time period of the present study, there was an increase in mammography coverage performed within the last two years from 71.1% in 2007 to 78% in 2018. This represented a growth rate of 0.741 per year (P < 0.001). Stratified according to the number of years of schooling, there was a linear trend of progression among women with 0 to 8 years of schooling, from 66.1% to 73.5% (P < 0.001), while the coverage among the other schooling-level ranges remained static. There was a tendency towards significant increases in coverage for all age groups, from 73.4% to 78.6% among women aged 50 to 59 years and from 67.2% to 76.9% for those aged 60 to 69 years. In all regions of Brazil, the trend was upward, and the northern region had the fastest growth rate (β = 1.613) among all the regions (Table 1).

**Table 1:** Temporal trends of mammography coverage among women (50 to 69 years old) over the last two years in the Brazilian state capitals and in the Federal District, according to sociodemographic characteristics. Vigitel; 2007 to 2018 (n = 385,255)

Variables		2007	2008	2009	2010	2011	2012	2013	2014	2015	2016	2017	2018	P-value	Angular coefficient (β)
	**Total**	71.1	71.7	72.4	73.4	74.4	77.4	78.0	77.8	78.1	78.2	78.5	78.0	< 0.001	0.741
**Education (years)**	0-8	66.1	66.5	66.4	67.5	67.8	71.4	72.9	71.8	71.9	71.2	72.3	73.5	< 0.001	0.709
	9-11	77.3	77.6	79.4	77.3	80.5	81.8	81.4	80.9	81.5	82.4	81.9	77.6	0.075	0.297
	≥ 12	87.6	88.8	87.9	87.8	87.6	90	88.3	91.8	89.3	90.5	87.3	87.9	0.457	0.092
**Age range**	50-59	73.4	74.2	74.1	75.9	77.3	79.7	79.6	78.8	79.8	78.0	79.9	78.6	< 0.001	0.558
	60-69	67.2	67.3	69.8	69.3	69.9	73.7	75.3	76.3	75.6	78.5	76.1	76.9	< 0.001	1.046
**Region**	North	60.2	59.0	60.3	63.7	64.4	70.7	70.9	70.9	72.6	77.5	72.4	74.4	< 0.001	1.613
	Northeast	71.6	71.6	70.9	71.9	72.6	76.9	77.1	76.4	77.4	77.6	78.3	76.2	< 0.001	0.688
	Center-West	72.3	70.0	69.6	79.2	72.9	73.4	79.6	78.2	79.6	79.2	79.6	75.6	0.014	0.748
	Southeast	70.9	73.0	74.5	73.4	75.7	78.2	78.3	78.4	78.3	77.8	78.9	79.2	< 0.001	0.700
	South	79.2	76.2	76.3	79.9	81.7	84.5	82.7	83.4	81.6	81.2	80.5	82.0	0.050	0.404

The coverage of cervical oncotic cytological testing performed within the last three years remained static, with 82.0% in 2007 and 81.7% in 2018. There were declining trends in coverage among women with 12 or more years of schooling (β = −0.463; P < 0.001) and among those aged 25 to 34 years (β = −0.356; P = 0.003). On the other hand, there was an increase in coverage among women aged 55 to 64 years (β = 0.402; P < 0.001). For all regions of Brazil, the coverage remained static (Table 2).

**Table 2:** Temporal trends of cervical oncotic cytological testing coverage among women (25 to 64 years old) over the last three years in the Brazilian state capitals and the Federal District, according to sociodemographic characteristics. Vigitel; 2007 to 2018 (n = 385,555)

Variables		2007	2008	2009	2010	2011	2012	2013	2014	2015	2016	2017	2018	P-value	Angular coefficient (β)
	**Total**	82.0	83.3	82.2	82.2	81.8	82.3	82.9	81.4	81.0	82.0	82.8	81.7	0.329	-0.055
**Education (years)**	0-8	78.6	80	77.7	78.7	77.6	78.3	78.3	77	77.8	76.7	79.5	79.3	0.692	-0.035
	9-11	83.7	83.7	83.1	81.3	81.6	81.7	83.7	81.1	80.1	82.6	83.0	80.1	0.086	-0.192
	≥ 12	87.9	90.2	89.2	89.7	88.5	88.5	87.3	86.2	84.9	85.9	85.4	84.8	< 0.001	-0.463
**Age range**	25-34	77.6	80.2	78.2	78.1	78.4	78.2	78.8	76.8	75.1	75.9	76.6	74.5	0.003	-0.356
	35-44	86.0	86.5	85.2	83.9	83.9	84.3	85.2	82.5	83.9	86.1	85.7	84.9	0.586	-0.057
	45-54	85.6	85.6	84.6	87.2	85.0	85.0	86.5	85.7	83.9	85.8	87.1	85.7	0.683	0.035
	55-64	78.2	80.5	81.7	80.5	80.5	83.5	81.8	82.5	83.3	82	83.8	84.1	< 0.001	0.402
**Region**	North	78.6	79.9	78.1	80.0	77.0	78.4	81.2	79.3	81.9	81.7	82.9	82.1	0.006	0.384
	Northeast	75.7	78.2	75.3	76.5	75.3	75.5	76.5	75.4	75	75.8	76.1	74.7	0.148	-0.114
	Center-West	80.3	79.3	80.8	78.3	78.6	81.5	79.5	79.1	77.6	79.8	80.2	79.4	0.645	-0.045
	Southeast	85.5	87.1	86.3	85.6	86.1	86.5	86.8	84.5	83.9	84.9	86.5	85.5	0.241	-0.099
	South	87.5	87.6	87.6	88.8	88.0	86.4	88.3	89.2	87.6	89.5	87.4	86.8	0.889	0.012

**Figure 1:**
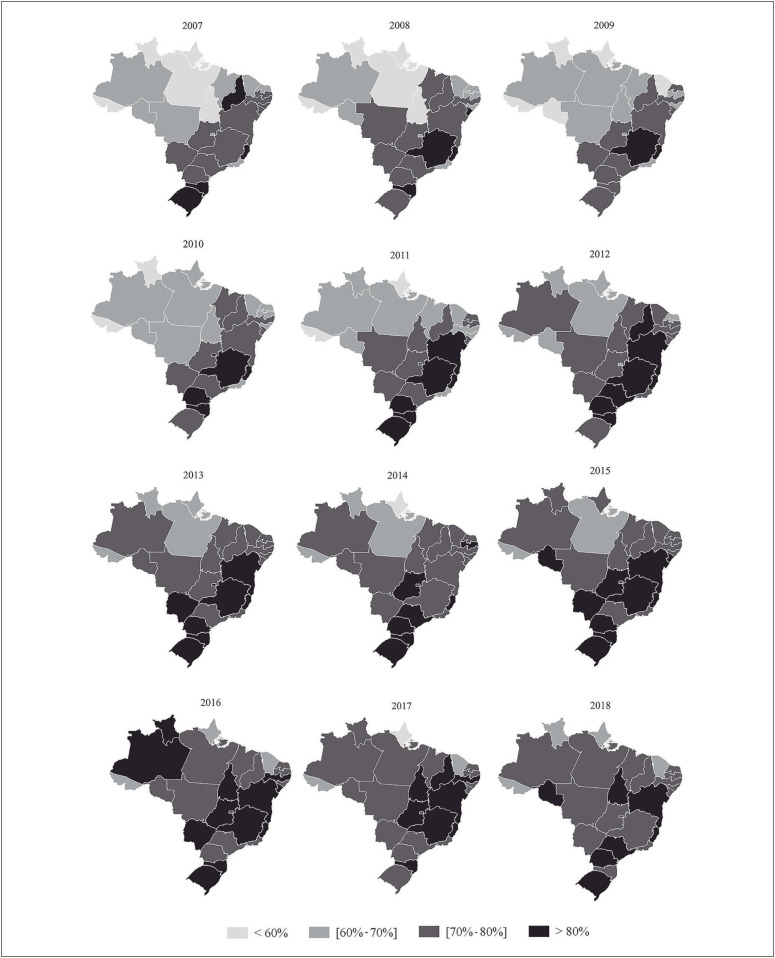
Frequencies of mammography coverage among women (50 to 69 years old) over the last two years in the Brazilian state capitals and in the Federal District. Vigitel; 2007 to 2018 (n = 385,555).

**Figure 2:**
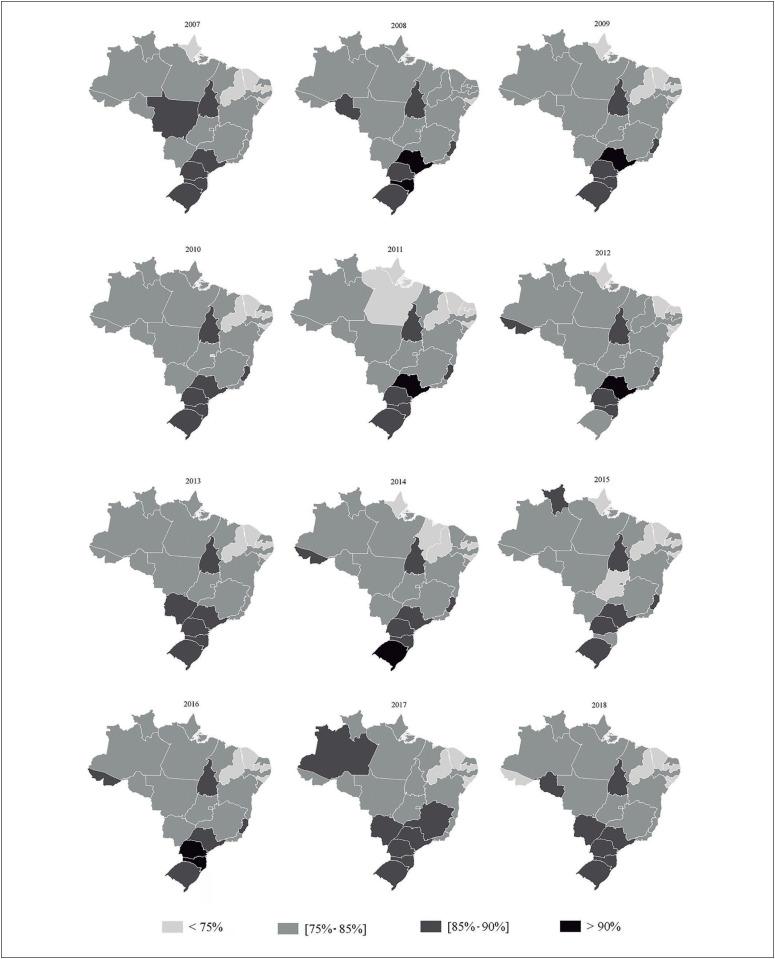
Frequencies of cervical oncotic cytological testing coverage among women (25 to 64 years old) over the last three years in the Brazilian state capitals and the Federal District. Vigitel; 2007 to 2018 (n = 385,255).

Figures 1 and 2 show maps of the distribution of mammography and cervical cytological testing coverage in all the Brazilian state capital cities and the Federal District. Mammography coverage above 70%, considering the entire period (2007 to 2018), was found in Aracaju, Belo Horizonte, Campo Grande, Curitiba, Florianópolis, Goiânia, Porto Alegre, Salvador, São Paulo, Teresina and Vitória (Figure 1). Regarding cervical oncotic cytological testing, coverage above 85% was only found in Curitiba, Palmas, Porto Alegre and São Paulo (Figure 2).

## DISCUSSION

This study showed that there were increases in mammography coverage between 2007 and 2018 among women with less education, for all age groups and regions of Brazil. Regarding cervical oncotic cytological testing, the tendency was for static coverage when considering the entire period. There were declining trends among women with 12 or more years of schooling and aged 25 to 34 years. The trends were upward among women aged 55 to 64 years and static for all regions.

The World Health Organization (WHO) advocates strategies for screening and early detection of cancer among women.^10^ Implementation of population-based breast cancer screening programs in developed countries has resulted in a 20% reduction in breast cancer morbidity and mortality.^10^ The Brazilian guidelines indicate mammography only for women aged 50 to 69 years, with two-year frequency.^1^ Mammography conducted among women aged 40 to 49 years presents lower detection sensitivity because of higher breast density at these ages, thus generating a greater number of false-positive results, with unnecessary exposure to radiation, surgical procedures and other events such as psychological distress and invasive examinations.^1^ Thus, the Brazilian Ministry of Health and the National Cancer Institute (INCA) contraindicate mammography in this age group, in the belief that the risks outweigh the benefits.^4^

There is no consensus regarding this contraindication among different countries and medical associations. In Brazil, the Brazilian Federation of Gynecology and Obstetrics (Federação Brasileira das Associações de Ginecologia e Obstetrícia, FEBRASGO) recommends that this screening test should be done annually in the 40 to 69 age group, which could explain the high number of women undergoing mammography under 50 years of age.^11^ On the other hand, the Swiss Medical Council does not recommend any mammographic screening program in any age group because it considers that the benefit is small and questionable.^4^

It is also noteworthy that the most recent evidence does not recommend breast self-examination, since its effectiveness has not been proven and health risks associated with this practice have been demonstrated.^1^ Analysis on data from the Global Burden of Disease study indicated that mortality remained stable from 1990 to 2015 in Brazil and its states. There was no significant increase in any of the states in the northern and northeastern regions.^12^ The increase in mammography coverage may explain the stability in mortality rates, but attention needs to be drawn to the worse performance in the northern and northeastern regions. Coverage was also lower in these regions and this resulted from uneven geographical distribution of mammography devices and the lower operational capacity in these locations.

Healthcare inequalities generate different exposures to factors that determine health, illness and death.^13^ It is important to advance in interventions on social determinants of health that require multisectoral and coordinated actions on the various aspects of life in different societies.^13^

Inequalities in the coverage of screening tests according to schooling level are socioeconomic determinants that can influence both the perception of risk and the behavioral factors that influence the decision to seek healthcare services. Such inequalities are of relevance with regard to access to these examinations.^14^

Although there were differences in mammography and Papanicolaou test coverage according to region and schooling level, these coverage levels were close to the targets set out in the national plan for combating noncommunicable diseases, i.e. 75 and 85% respectively.^7^ These findings are a reflection of the implementation of several policies, programs, actions and strategies by the Ministry of Health over the last decade, with emphasis on the National Policy for Comprehensive Care for Women's Health, the National Policy for Oncological Care and the Plan for Strengthening the Cancer Prevention, Diagnosis and Treatment Network, which included the National Cervical and Breast Cancer Control Program and the Strategic Action Plan for Combating Chronic Noncommunicable Diseases in Brazil, 2011-2022.^15^ The expansion of primary care actions and the More Doctors Program (Programa Mais Médicos) were essential for expanding the provision of actions relating to women's health and controlling cervical and breast cancers.^12^

The importance of advancing communicative and preventive actions, especially among women with lower schooling and income levels in the poorest state capitals of the country needs to be highlighted. Such actions have the aim of increasing the frequency with which women undergo examinations and their adherence to examination programs.^3^ These programs, policies and actions aimed at improving women's health, together with the expansion of primary care, have also enabled greater access and knowledge of these tests for all women, regardless of income, schooling and race, thus also reducing healthcare inequalities.^3^ Therefore, expanding investment in SUS is one of the solutions for reducing social disparities, and this can be understood to be a policy for reducing inequities.^17^

The results from this study present some limitations. Self-reported data collected through telephone interviews are subject to the potential for information bias. Moreover, the Vigitel results refer to the adult population living in the 26 Brazilian state capitals and the Federal District and, therefore, these results cannot be extrapolated to the entire Brazilian population. Another limitation relates to the concept of Papanicolaou test coverage. The samples need to be satisfactory and, for the coverage to be considered adequate, the initial screening must take place with two negative examinations with a one-year interval between them, so that it becomes possible to move on to examinations every three years. These data regarding the sample and two negative results with a one-year interval were not addressed in the Vigitel questions during the telephone interview because of the specificity of the desired responses. In Brazil, obtaining access to the information needed for cervical cancer screening to be considered ideal is a challenge, given that there are no adequate surveillance mechanisms and no monitoring of the coverage of these tests. Papanicolaou examinations in Brazil are conducted in an opportunistic manner, and not through an organized scheme of surveillance and monitoring.

## CONCLUSION

There was a trend of increasing mammography coverage among women aged 50 to 69 years and a static trend regarding cervical oncotic cytological testing among women aged 25 to 64 years living in Brazilian state capitals and the Federal District. However, differences in prevalence were observed, such that it was higher among better educated women and among women living in the southern and southeastern regions. Therefore, there is still a need to expand the provision, quality and availability of actions and services aimed at improving women's health and, above all, to prioritize investments in the regions that had the least coverage of these tests.
